# Ectoparasites infestation of free-ranging hedgehog (*Etelerix algirus*) in north western Libya

**Published:** 2014-02-20

**Authors:** M.M. Hosni, A.A. El Maghrbi

**Affiliations:** 1 *Department of Preventive Medicine, Faculty of Veterinary Medicine, University of Tripoli, P. O. Box 13662, Tripoli, Libya*; 2 *Department of Microbiology and Parasitology, Faculty of Veterinary Medicine, University of Tripoli, P. O. Box 13662, Tripoli, Libya*

**Keywords:** Ectoparasites, *Etelerix algirus*, Hedgehogs, Libya

## Abstract

The aim of this study was to assess the prevalence of ectoparasites in hedgehogs (*Etelerix algirus*) in north western region of Libya. Seventy hedgehogs were sampled, and 39 (55.7%) were infested with external parasites. A total of 44 ticks, 491 fleas were collected from the infested hedgehogs and four species of ectoparasites were identified, one mite (*Sarcoptes scabiei*), one tick (*Rhipicephalus appendiculatus*) and two fleas (*Xenopsylla cheopis* and *Ctenocephalides canis*). For ectoparasites, 10/39 (25.6%) were infested by *S. scabiei*, 8/39 (20.5%) by *Rh. appendiculatus* and 11/39 (28.2%) by fleas. The prevalence of mixed infestation with *S. scabiei* and *C. canis* was 3(7.7%), *Rh. appendiculatus* and *C. canis* was 2 (5.1%) and infestation by two species of fleas was 5 (12.8%). The overall mixed infestation was 10 (25.6%). We concluded that the hedgehogs may play an important role in spreading external parasites and transmission of diseases from one region to another and from wildlife animals to domestic animals and human.

## Introduction

Desertification has altered the ecological dynamics in the sub-Saharan region. Deforestation and human induced landscape alteration observed in Libya over the last thirty years, has led to the immigration of wild animals from their natural geographical zones to areas inhabited by humans and domestic animals. Therefore, small mammals such as hedgehogs, fox and hares have become synanthropic species and are subsequently exposed to new pathogens in a new environment. There are little available data regarding the species, population and geographical distribution of hedgehogs (Family Erinaceidae) in Libya; consequently the diseases that occur in this animal are also not well studied and documented.

Hedgehogs are hosts for a wide variety of parasites, bacteria, viruses and fungi and they can play a significant role in the transmission dynamics of some zoonotic pathogen (McCarthy and Moore, 2000; Riley and Chomel, 2005). Hedgehogs are ground foraging mammals seeking invertebrates and small vertebrates, and are naturally exposed to haematophagous ectoparasites (Dziemian *et al.*, 2010).

Hedgehogs can carry several ticks and fleas species; the load of these ecoparasites can vary among individuals and parasitization rates of hedgehogs in urban environments can be affected by heterogenous landscape matrices effects (Thamm *et al.*, 2009). *Sarcoptes scabiei* has been reported from more than 100 species of domestic and wild animals (Bak *et al.*, 1997). The objective of the present study was to survey ecoparasites infesting free-ranging hedgehogs in north western region of Libya.

## Material and Methods

### Study area

The study was carried out in the north western region of Libya. This area receives an average annual rain fall (50-250 mm); winter is the main season for rainfall which is concentrated in the coastal strip declining in amount and frequency towards the south. The ambient temperature is generally ranges from mild to cold in winter (4-24°C) and hot in late spring and summer (15-44°C).

**Fig. 1:**
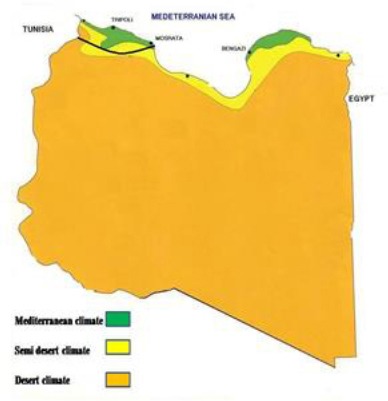
Map of Libya illustrating study area (black line), and climate decline from north to south.

Vegetation in the study area is more lush in north and increasingly arid towards the south, changing first into scant grazing and finally to sub-Saharan areas toward the south (Fig. 1). This area is considered to be a grazing area for Libyan livestock which includes more than 50% of five million sheep and goats, and about 109,397 of camels (LGIA, 2007). It is also inhabited by several species of animals including some endangered species as *Gazella dorcas* and *Vulpes zerda* (FAO, 1992).

### Collection of samples

During the period from June 2008 to December 2009, seventy free-ranging hedgehogs (*E. algirus*) were captured live from farms and roadsides at night by hand using spotlights and protective gloves. Hedgehogs were transported in cages from their natural habitat to the laboratory where they were kept and fed in individual cages. For parasite sampling, each hedgehog was anaesthetized using a piece of cotton soaked in diethyl ether and then carefully examined for external parasites and skin lesions.

After the examination and parasite collection, animals were returned to their sites of capture and released. The collected ticks and fleas were immediately preserved in labeled glass vials containing 70% alcohol and glycerin.

Ticks and fleas were prepared and examined microscopically and identified to species according to Engelbrecht *et al*. (1965). Where mange-like lesions were present; the spines or hair around affected area were removed and a skin scraping was made with a scalpel (Soulsby, 1982).

All collected ectoparasites were identified to species level according to the identification key of Baker *et al*. (1958), Soulsby (1982) and Hoogstraal and Kaiser (1958).

## Results

**Fig. 2:**
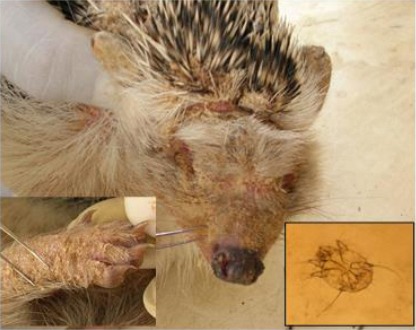
Hedgehog (*E. algirus*) with mange (*S. scabiei*) on face and leg.

**Fig. 3:**
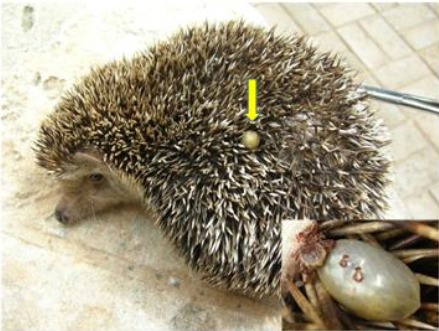
Hedgehog (*E. algirus*) infested with ticks (Male and female).

Seventy hedgehogs were sampled in total, 39 (55.7%) of which were found to be infested with ectoparasites (Table 1). Four species were identified, one mite (*Sarcoptes scabiei*) (Fig. 2), one tick (*Rhipicephalus appendiculatus*) (Fig. 3) and two fleas species (*Xenopsylla cheopis* and *Ctenocephalides canis*). A total of 44 ticks and 491 fleas were collected from infested hedgehogs. The prevalence of *S. scabiei, Rh. appendiculatus* and the two species of fleas were 10 (25.6%), 8 (20.5%) and 11 (28.2%) respectively.

**Table 1:** Prevalence rate of ectoparasites infestation found in hedgehogs in north western Libya.

Hedgehog (*E. algirus*)		Mites	Ticks	Fleas		Mixed infestation*		
No. of animal examined	No. of animal infested	*Sarcoptes scabiei*	*Rhipicephalus appendiculatus*	*Xenopsylla. Cheopis*	*Ctenocephalides canis*	*Sarcoptes scabiei* *Ctenocephalides canis*	*Rhipicephalus appendiculatus* *Ctenocephalides canis*	*Xenopsylla. Cheopis* *Ctenocephalides canis*
70	39 (55.7%)	10 (25.6%)	8 (20.5%)	3 (7.7%)	8 (20.5%)	3 (7.7%)	2 (5.1%)	5 (12.8%)

* Overall mixed infestation was 10 (25.6%).

The prevalence of mixed infestation with *S.scabiei* and *C. canis* was 3 (7.7%), *Rh. appendiculatus* and *C. canis* was 2 (5.1%), and infestation by two species of fleas was 5 (12.8%).

## Discussion

The present study showed that 39 (55.7%) out of 70 hedgehogs were infested with arthropods. The prevalence rate of mite *S. scabiei* infestation in the present study was 25.6%. However, other authors found lower rate with 15.4% in *E. algirus* (Hosni, 2006). Investigation of the African hedgehogs (*A. albiventris*) in Nigeria revealed the occurrence of *S. scabiei* (Okaeme and Osakwe, 1985). *S. scabiei* was also reported on the red foxes (*Vulpes vulpes*) by Morner (1992), Forrester (1992) and Ahmed (1994). Fleas (*X. cheopis* and *C. canis*) were reported in this study with a prevalence of (28.2%).

Hosni (2006) recorded a lower prevalence with (17.3%) in *E. algirus* in Libya. Visser *et al*. (2001*)* in Germany recorded three species of flea in hedgehog (*Archaeopsylla erinacei*, *Ceratophyllus gallina* and *C. felis*). In a previous study, Cerqueira *et al*. (2000) also collected *X. cheopis* from the red fox (*Vulpes vulpes*) which is in overlapping distribution with the hedgehogs in this area.

In addition, Hosni (2006) reported that common jackal (*Canis aureus*) was infested with ectoparasites (*X. cheopis*, *S. scabiei* and *Rh. appendiculatus*). Only one tick species *Rh. appendiculatus* was detected in the present work with a prevalence of 20.5% supported by a similar finding by Hosni (2006). Khaldi *et al*. (2012) found that 77.7% and 91% of hedgehogs (*A. algirus*) examined were infested with fleas (*A. erinacei*) and ticks (*Haemophysalis erinacei and Rh. sanguieus*) in Algeria.

Higher levels of ectoparasitism were reported in wild animals (67%) rather than domestic stock (39%) in the Province of Burgos, Spain by Domínguez-Peñafiel *et al*. (2011). Over a third of the animals (39%) in this study were infected by ticks with *Ixodes ricinus* and *Ixodes hexagonus* as the most prevalent species. The overall prevalence of parasitism by fleas was 27%.

The ectoparasites of domestic animals in Libya have been examined previously in a number of studies. A flea infestation of farm animals in northern Libya was reported where 12,130 sheep examined from 124 flocks, and 150 sheep were found to be infested with fleas from 50 different flocks. 1574 specimens of *Ctenocephalides felis strongylus* and 4 specimens of *Pulex irritans* were collected from a sheep (Kaal *et al.*, 2006). In other studies, 2287 farm animals (cattle, camels, sheep, goats, horses, donkeys, dogs and rabbits) suspected of carrying parasitic mites were examined at 58 farms throughout Libya. Mites were identified on 1303 of these animals. The most common parasites on cattle were *Psoroptes* and *Chorioptes*, on camels and sheep were *Sarcoptes* and *Psoroptes*, goats were *Sarcoptes* and *Demodex*, horses were infested with *Psoroptes* or *Chorioptes*, and one donkey carried *Sarcoptes*. *Otodectes* was common on dogs. Rabbits often had psoroptic ear mange or sarcoptic body mange (Gabaj *et al.*, 1992).

One thousand three hundred sixty six sheep examined for a period of one year to determine common ectoparasites in eight localities in Libya. They found that 430 (31.48%) sheep were infested. The species identified as *C. canis, Linognathus africanis, S. scabiei, Psoroptes ovis, Hy. Marginatum* and *Rh. appendiculatus* (Rashed *et al.*, 2010).

Evidently, there are a number of parasites in the areas which hedgehogs are recently coming into contact with. A study on occurrence of *Trichinella* spp. in wild animals in northwestern Libya was carried out by Hosni *et al*. (2013).

The results of this study indicate that the free-ranging hedgehogs (*E. algirus*) can serve as a source or a sink of several ectoparasites. They may, therefore, play a role in disease transmission to domestic animals, wildlife and humans. Hence, more research is needed to further investigate the hedgehog ectoparasites infestations and their role in the transmission of diseases in Libya.
